# Stromal vascular fraction cells as biologic coating of mesh for hernia repair

**DOI:** 10.1007/s10029-020-02135-4

**Published:** 2020-02-24

**Authors:** O. Guillaume, B. Pérez-Köhler, B. Schädl, C. Keibl, N. Saxenhuber, P. Heimel, E. Priglinger, S. Wolbank, H. Redl, A. Petter-Puchner, R. Fortelny

**Affiliations:** 1 3D Printing and Biofabrication Group, Institute of Materials Science and Technology, TU Wien, Vienna, Austria; 2 Austrian Cluster for Tissue Regeneration, Vienna, Austria; 3 Department of Medicine and Medical Specialties, University of Alcalá, Madrid, Spain; 4 Biomedical Networking Research Centre On Bioengineering, Biomaterials and Nanomedicine (CIBER-BBN), Madrid, Spain; 5 Ramón y Cajal Health Research Institute (IRYCIS), Madrid, Spain; 6 Ludwig Boltzmann Institute for Experimental and Clinical Traumatology, Vienna, Austria; 7 University Clinic of Dentistry, Medical University of Vienna, Vienna, Austria; 8 Department of General, Visceral and Oncologic Surgery, Wilhelminenspital, Vienna, Austria

**Keywords:** Stromal vascular fraction, Adipose tissue, Mesh, Abdominal hernia, Angiogenesis

## Abstract

**Background:**

The interest in non-manipulated cells originating from adipose tissue has raised tremendously in the field of tissue engineering and regenerative medicine. The resulting stromal vascular fraction (SVF) cells have been successfully used in numerous clinical applications. The aim of this experimental work is, first to combine a macroporous synthetic mesh with SVF isolated using a mechanical disruption process, and to assess the effect of those cells on the early healing phase of hernia.

**Methods:**

Human SVF cells combined with fibrin were used to coat commercial titanized polypropylene meshes. In vitro, viability and growth of the SVF cells were assessed using live/dead staining and scanning electron microscopy. The influence of SVF cells on abdominal wall hernia healing was conducted on immunodeficient rats, with a focus on short-term vascularization and fibrogenesis.

**Results:**

Macroporous meshes were easily coated with SVF using a fibrin gel as temporary carrier. The in vitro experiments showed that the whole process including the isolation of human SVF cells and their coating on PP meshes did not impact on the SVF cells’ viability and on their capacity to attach and to proliferate. In vivo, the SVF cells were well tolerated by the animals, and coating mesh with SVF resulted in a decrease degree of vascularity compared to control group at day 21.

**Conclusions:**

The utilization of SVF-coated mesh influences the level of angiogenesis during the early onset of tissue healing. Further long-term animal experiments are needed to confirm that this effect correlates with a more robust mesh integration compared to non-SVF-coated mesh.

## Introduction

Soft tissue prolapses including abdominal wall hernia and pelvic organ prolapses are becoming a major problem in our society. Close to 30% of the population, above 60 years old is affected by soft tissue prolapses and around 1000,000 of prostheses are implanted annually in USA for hernia repair. Utilization of synthetic polymers such as polypropylene (PP) meshes has rapidly spread since 1950’s up to now and is still the “gold standard” for soft tissue reinforcement [1]. Nevertheless, macroporous PP meshes are still responsible for complications, and researchers quest for mesh improvement, by combining for examples meshes with therapeutics or cells [2, 3]. One emerging and promising experimental approach for challenging elective procedures in hernia surgery is to combine cell-based regenerative medicine to the mesh materials, and among all patient’s own cells [4]. The available literature conducted on preclinical models tends to demonstrate that seeding mesh implant with autologous cells prior to implantation improves globally the biocompatibility of the biomaterials, in terms of tissue adhesion, tissue remodeling, and graft vascularization [5–7]. However, there are several clinical drawbacks concerning the utilization of autologous fibroblasts or myoblasts in term of time, cost, safety, and regulation. Moreover, due to limitation of patient’s cell availability, development of stem cell therapy for soft tissue repair has emerged over the last few years [4, 8].

In stem cell-based therapies, stromal vascular fraction (SVF) isolated from adipose tissue following liposuction is at the center of intense research [9]. SVF represents a heterogeneous population of different stem cells (mesenchymal stromal cells (MSCs), hematopoietic stem cells, etc.), as well as endothelial progenitor cells and mature cells (adipocytes, fibroblasts, endothelial cells, smooth muscle cells, etc.) [10–12]. MSCs count only for 1 in 100,000 cells in bone marrow, whereas 2% of lipoaspirate cells are stem cells. In addition, those cells are relatively easily available without further need for expansion on plastic plates (minimizing risk of infection and mortality of the cells). Clinical trials have shown great potential of SVF cells, for instance in orthopedic surgery. This is illustrated by the recent publication from Coughlin et al*.*, in June 2017, reporting the harvesting procedure of SVF cells from abdominal liposuction (using minimal invasive procedure) for the treatment of degenerative joint disease of the knee [13]. Nevertheless, to the best of our knowledge, such translation to the field of abdominal wall hernia repair has not been reported so far. Our hypothesis is that the transplantation of SVF obtained using an enzyme-free-based protocol, combined with synthetic mesh impact on the early healing stage of the mesh, in terms of inflammation, angiogenesis, and peri-prosthetic tissue deposition.

## Materials and methods

### Liposuction procedure and isolation of SVF cells

The collection of human adipose tissues was approved by the local ethical board with patients’ written consent. SVF cells were isolated from adipose tissue via mechanical disruption and purified using centrifugation at 1200*g* for 7 min, followed by washing step of the SVF pellets with PBS. Finally, the resulting SVF cells were counted and their viability was assessed using a Cellometer Auto 2000 cell viability counter. The volume of fat processed was 300 mL and 150 mL for the in vitro and in vivo experiments, respectively.

### In vitro cultivation and characterization

#### Fabrication of the SVF-coated mesh

Aliquots of SVF, containing 400,000 cells, were resuspended in 100 µL of PBS and mixed in a solution of 25 µL of fibrinogen (ARTISS Sealant, Baxter Healthcare Corporation). After mixing, 50 µL of the thrombin solution was added and the reconstituted cell suspension (final volume of 175 µL) was quickly pipetted onto the surface of 1-cm^2^ mesh (TIMESH from pfm-medical AG, Köln, Germany), which is a lightweight polypropylene titanized mesh of a density of 35 g/m^2^.

#### In vitro cultivation, viability test, and microscopic observation

SVF-coated meshes were incubated in EGM-2 medium supplemented with aprotinin to inhibit fibrinolysis (at a final concentration of 100 KIU/mL). Viability of the cells was assessed through live/dead staining. The viable and dead cells were monitored using green and red fluorescence, respectively. Cells growing in the SVF-coated meshes were observed using scanning electron microscopy (JOEL JSM-6510 SEM). This requires first a fixation of the cells using 4% formaldehyde solution and a gradual dehydration with ethanol up to 100% followed by immersion in hexamethyldisilazane. Finally, after complete drying under a fume hood, the cell-coated meshes were sputter coated with Pd–Au and visualized with the SEM at 10 kV.

### Animal experiment

The project was approved by the ethical review board of the City of Vienna. Male athymic nude rats, weighing around 420 g (Envigo, Italy) were used for this project. The rats were anaesthetized by inhalation of oxygen with 1–2% isoflurane. Pain-killer treatment was administrated, composed of 1 mg/kg meloxicam, Metacam® *per os* 2 h before operation and 4 days after operation. To reduce animal suffering, the rats additionally received 0.05 mg/kg of buprenorphine (Bupaq®) subcutaneously every 6 h during the first 48 h.

#### Surgical procedure and euthanasia

**Fig. 1:**
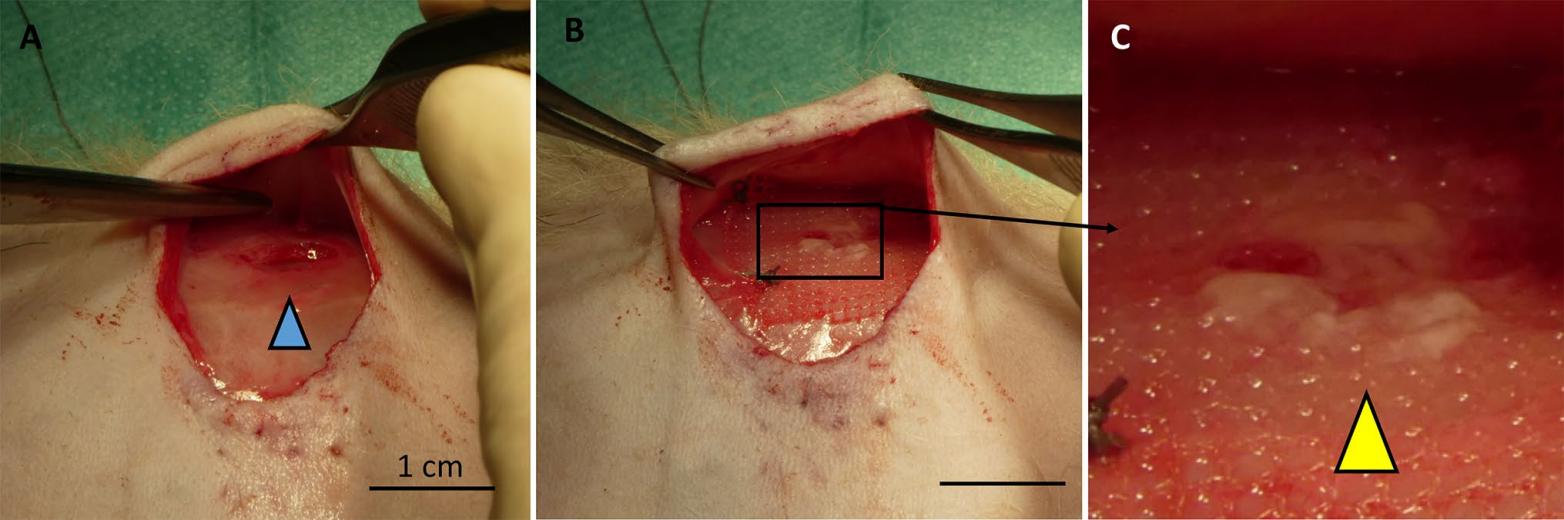
Onlay model used to evaluate the healing response of SVF-coated mesh. Hernia defects of 0.5 × 0.5 cm^2^ were created on each side of the linea alba of the abdominal wall of nude rats (defect is shown with the blue arrow, **a**). The created defects were either covered with SVF-coated mesh or with cell-free fibrin-coated mesh as control group (the yellow arrow denotes the SVF covering the hernia defect, **b** and **c**). Scale bars represent 1 cm in **a** and **b**

Onlay model was performed with creation of two muscular defects of 0.5 × 0.5 cm^2^ on each side of the linea alba, as previously presented [14]. The hernia defects were then repaired using either a SVF-coated mesh or a cell-free fibrin-coated mesh (used as control), randomly attributed to either the right or to the left side of the abdominal area (*n* = 14 per group) (the model is illustrated Fig. 1). The meshes were stabilized with four stitches on each corner and the skin of the animals was sutured. After 10 or 21 days, the animals (*n* = 7/time points) were sacrificed in deep anesthesia with first 100 mg/kg ketaminhydrochlorid (Ketasol®) and 12 mg/kg xylaxin (Rompun®) intraperitoneal, and then thiopental natrium intracardial.

### Biological response of the meshes

#### Macroscopic observation

The degree of the mesh integration in the abdominal tissue and of the mesh shrinkage was quantified from 0 to 100%. The presence of dislocation, seroma, and the intensity of the mesh vascularization, and inflammation were also reported.

#### Doppler scanning

Micro-vascular blood flow was estimated with a Laser Doppler Imager (LDI, Moor instruments Ltd) in the 21-day follow-up group. Shortly before the euthanasia, the animals were placed under the Laser Doppler Imager to scan the area of the operated abdominal wall. Flux values were transferred to a color scale, visualizing the blood flow of the grafts and further processed for quantification.

#### High-resolution computer tomography

Barium sulfate (Micropaque, Guerbet GmbH) was used to visualize vascular structures via high-resolution microcomputed tomography (microCT). Micropaque was diluted to 30% with 2% (*w*/*v*) porcine gelatin at 40–50 °C and applied as a full-body perfusion. Prior to contrast agent perfusion, fixation was performed by first a perfusion with a heparin saline solution, 10 IU/mL followed by a 4% formaldehyde solution. Finally, saline solution was perfused before the contrast agent was applied. Each perfusion step was performed for 5 min upon which animals were stored on ice to harden the micropaque gelatin solution. Samples were then excised and, to increase the contrast between the tissue and the mesh, the grafts were finally incubated in a solution of potassium iodide at 1%. Finally, the samples were scanned with a high-resolution CT scanner (Scanco 50, Scanco Medical AG, Swiss) at 90 kV, 200uA in 1000 projections using a voxel size of 17.2 µm.

#### Histology and immunohistology

All grafts explanted after the euthanasia were fixed in 4% buffered formaldehyde solution and, after 24 h, were washed and then dehydrated in 50% ethanol for 1 h, and were then preserved in a solution of 70% ethanol. Finally, samples were embedded in paraffin, cut using microtome and stained with hematoxylin and eosin (H&E).

Anti-human mitochondrial immunostaining was performed using HIER (heat-induced antigen retrieval), in EDTA buffered pH 9, followed by incubation with a mouse monoclonal anti-human mitochondria antibody.

A blinded analysis of the histological slides was performed by a histopathologist to assess the tissue integration of the meshes, in terms of connective tissue, graft vascularization, and foreign-body reaction. For each sample, the density of the fibers present in the neoformed connective tissue surrounding the implant was measured according to the following grading scale system (1-very loose; 2-loose; 3-dense; 4-very dense). Grafts vascularization was assessed by performing counts of the vessel cross sections observed per field (six fields taken at random location). Foreign-body giant cells (FBGC) were also quantified (three fields taken at random location) to evaluate the foreign-body reaction of the different grafts. All these measurements were carried out using ImageJ picture processing and analyzing software.

### Statistical analysis

All the statistical analyses were performed using GraphPad Prism 5 software for Windows (GraphPad Software, Inc., La Jolla, CA, USA). Results are expressed as mean ± standard deviation. The Student’s *t* test was used with a significance level of *p* < 0.05.

## Results

**Fig. 2:**
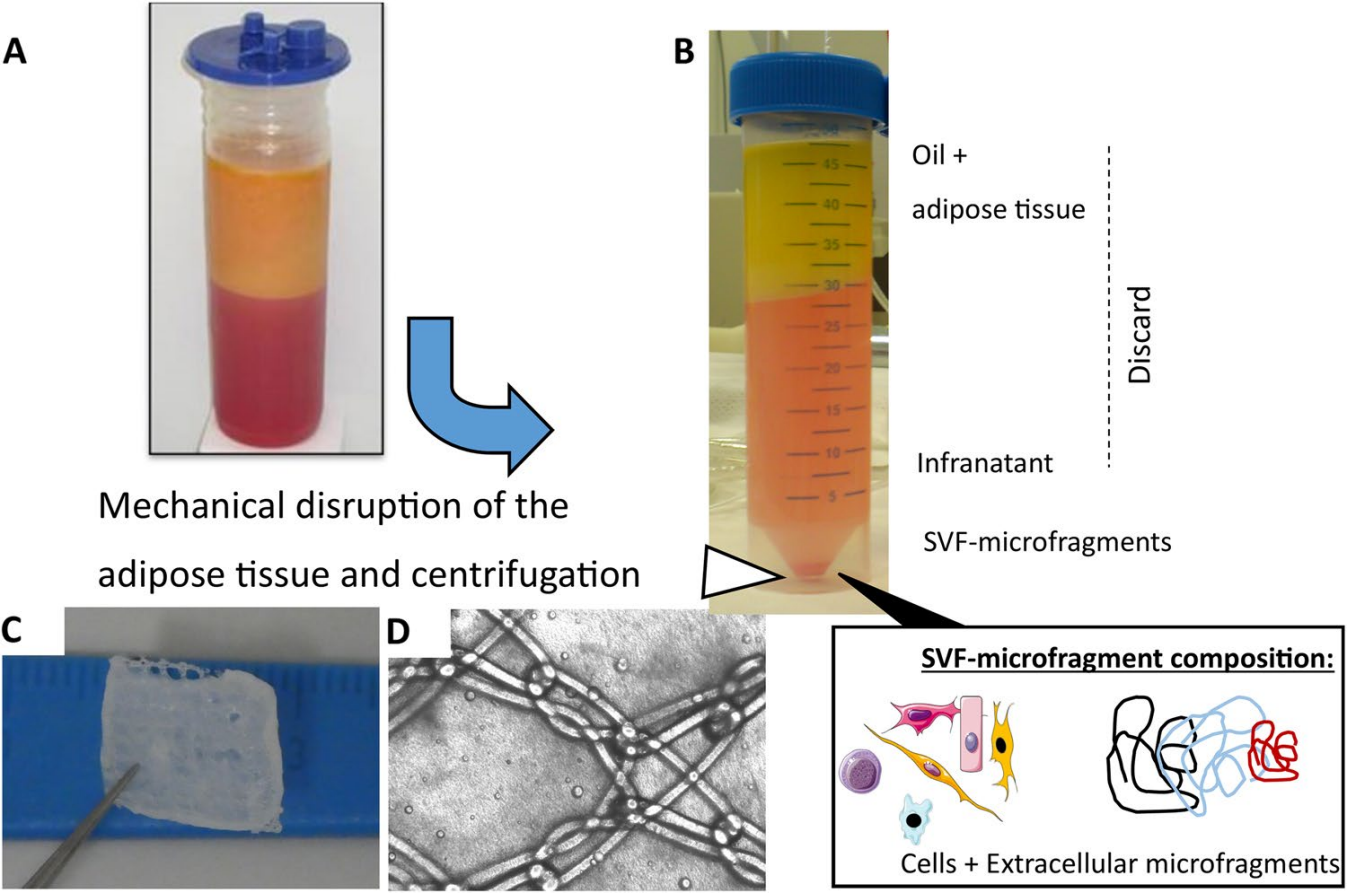
Processing fat tissue and mesh coating. Following liposuction, adipose tissue is processed through mechanical disruption in sterile condition (**a**). After centrifugation of the resulting fluid at 1200*g* for 7 min, only the SVF was collected (**b**), and subsequently washed. SVF cells were mixed with fibrin gel and used to cover the surface of a synthetic macroporous mesh (**c** and **d**)

**Fig. 3:**
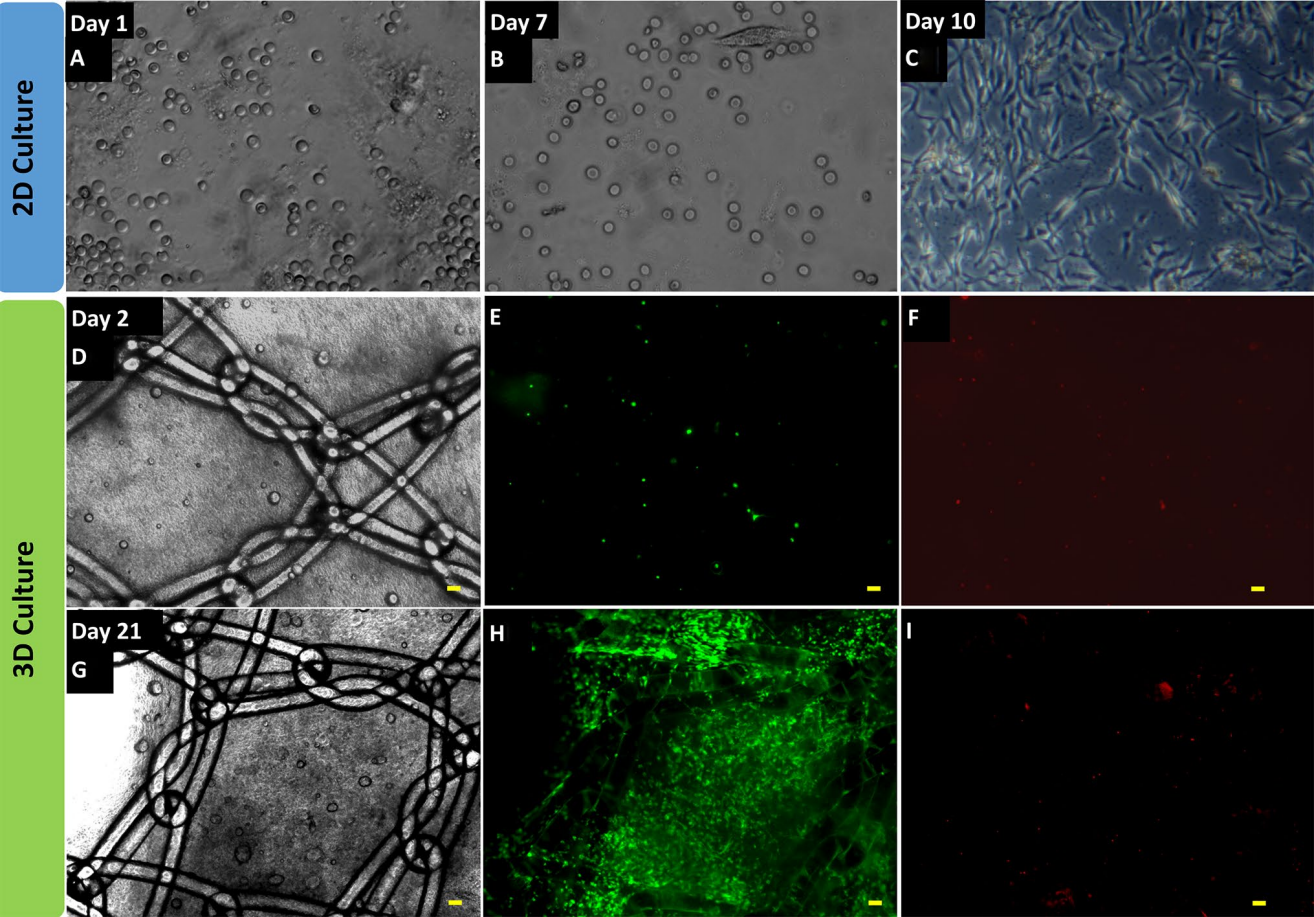
SVF cell behavior on 2D and 3D culture systems and viability over time. Illustration of the SVF cell adhesion kinetic at day 1, 7, and 10 (**a**, **b** and **c,** respectively) after plating onto tissue-culture polystyrene (two dimension). Appreciation of the cell viability in vitro using Live/Dead staining after coating 1 cm^2^ of mesh with 400,000 cells (three dimension), at day 2 (**e** and **f**) and 21 (**h** and **i**). Corresponding bright-field views are shown in **d** and **g** for day 2 and 21, respectively. Living cells appear in green fluorescence (**e** and **h**) and dead cells appear in red fluorescence (**f** and **i**). Scale bars represent 100 µm

After processing the liposuction tissues, the SVF cells still embedded in their native extracellular matrix (ECM) microfragments can easily be separated from the waste tissues (Fig. 2a, b). The resulting SVF viability was ranged between 75 and 80%, which is close to other reports employing enzymatic methods [11, 15]. The obtained SVF can then be integrated to a mesh coating through the use of a degradable and biocompatible hydrogel carrier, in our case fibrin clot (Fig. 2c, d). Once processed through mechanical disruption, the SVF cells exhibited an initial round phenotype (non-adhering cells, Fig. 3a, b) and were then able to adhere and proliferate until confluency on the tissue culture plastic plates in 7–10 days (cultivated in two dimension, Fig. 3c). In three dimension, after 2 days of incorporation in a fibrin coating covering PP mesh (Fig. 3d), the SVF maintained a great viability (the majority of the cells appeared in green fluorescence upon live/dead staining, Fig. 3e, f) and exhibited as well a round-shape phenotype as described previously for 2D condition. After 21 days, the number of cells increased dramatically (Fig. 3g), which can be seen by the strong increase of the green fluorescence intensity (Fig. 3h). At such late time point, the majority of the cells has been able to adhere onto and to infiltrate into the fibrin clot.

**Fig. 4:**
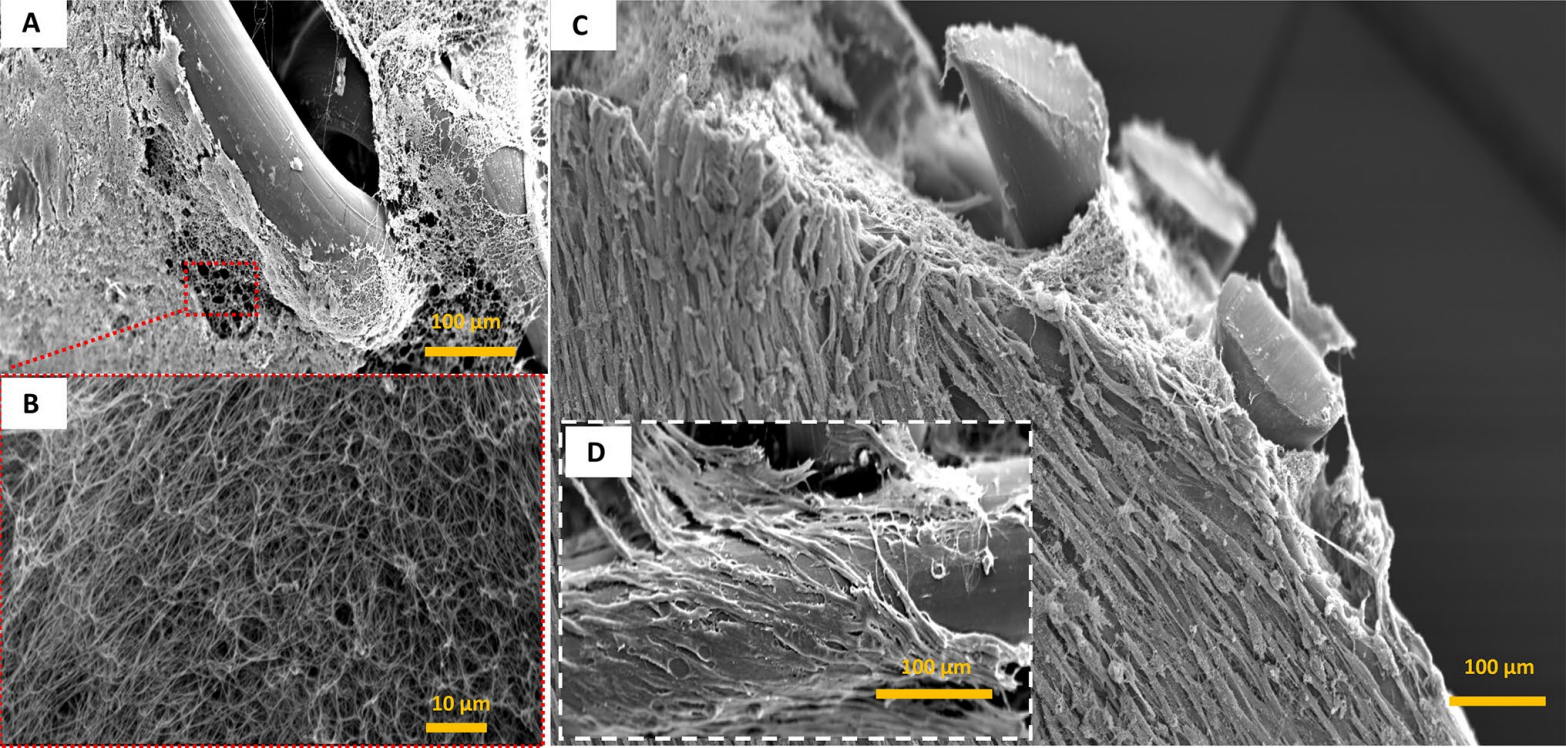
Scanning electron microscopy (SEM) imaging of the SVF-coated meshes. SEM was conducted at day 2 (**a** and **b**) and at day 21 (**c** and **d**) after SVF seeding. Scale bars represent 100 µm for pictures **a**, **c** and **d** and 10 µm for picture **b**

The limited number of cells presents at early time point in the 3D culture system (i.e. the fibrin-coated mesh) is also visible using SEM imaging (Fig. 4). Indeed, after 2 days, only fibrin microfibers were observed surrounding the filaments of the PP mesh (Fig. 4a, b). The intense cellular proliferation was clearly shown at a later time point (day 21, Fig. 4c), with numerous cells spreading onto and into the fibrin clot and adhering on the surface of the mesh filaments (Fig. 4d). To assess in vivo the potential of such biological coating, we covered the synthetic PP meshes with human SVF and implanted the grafts in an onlay model using immunodeficient rats (to prevent any immune rejection of the human SVF, model shown Fig. 1). During the 21 days of the experiment, no sign of suffering or adverse effects related to the surgical act or to the biomaterials implanted were detected for both the SVF-free group and the SVF-coated group. The results of the macroscopic observations monitored at the euthanasia of the rats at day 10 and day 21 are shown in Table 1.

**Table 1:** Morphological assessment of the macroscopic mesh integration and other complications at 10 days and 21 days post-operatively

Macroscopic observation	Day 10 SVF group	Day 10 Fibrin group	Day 21 SVF group	Day 21 Fibrin group
Mesh shrinkage (in %)	0, 30, 0, 0, 0, 0	0, 0, 0, 0, 0, 0	0, 0, 0, 0, 0, 0, 0	0, 0, 0, 0, 0, 0, 0
Tissue integration (in %)	100, 100, 100, 100, 100, 100	100, 100, 100, 100, 100, 100	100, 100, 100, 90, 100, 100, 100	85, 100, 90, 100, 100, 100, 100
Complications	None	None	None	None

No major complications were noted for both groups and the meshes were well integrated after 10 and 21 days. Only one case of mesh shrinkage of 30%, compared to the initial 4-cm^2^ surface of the graft was measured for the SVF group at day 10.

**Fig. 5:**
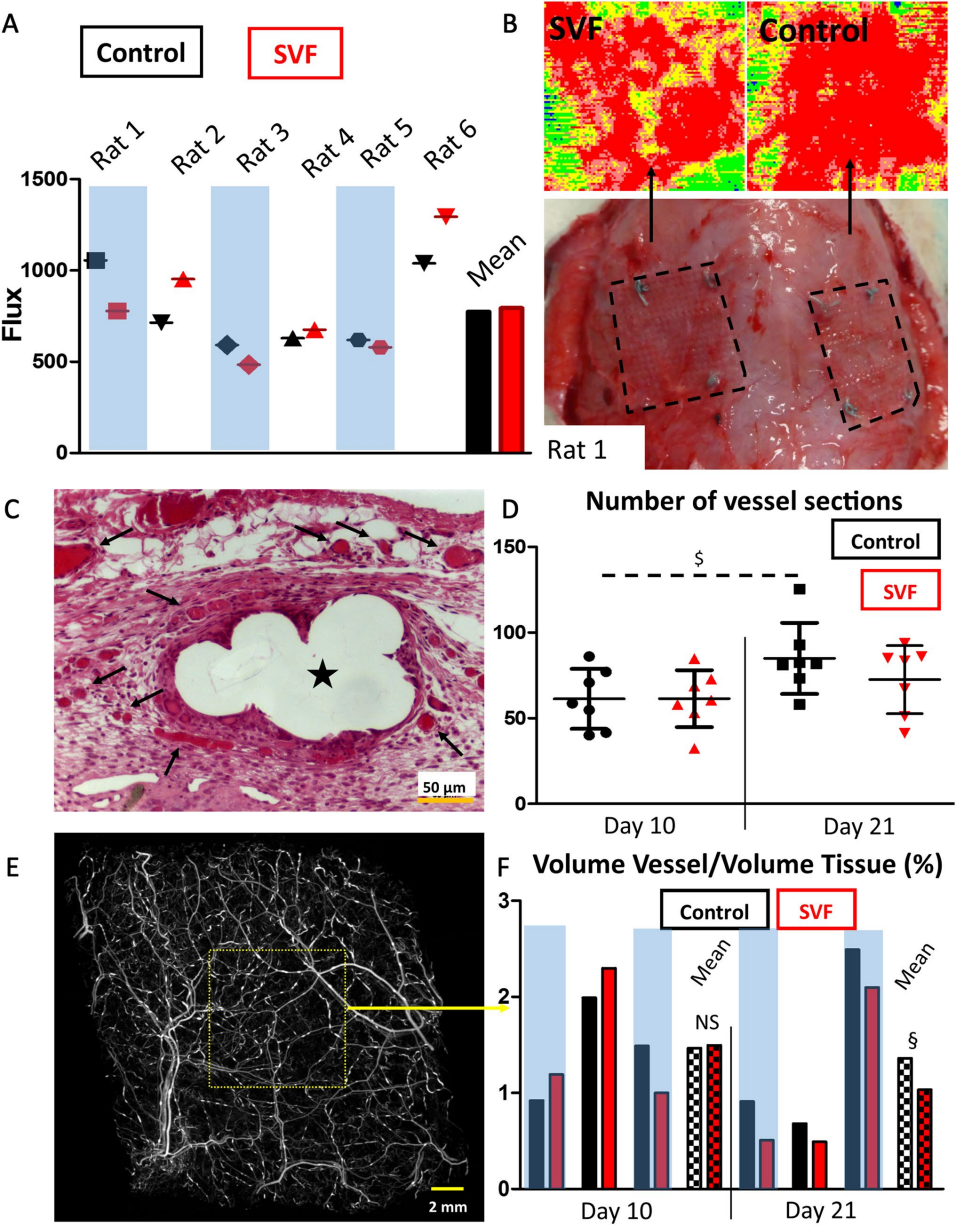
Effect of SVF coating on the degree of vascularization. Doppler analysis of the mesh vascularization conducted after 21 days of implantation for control (in black symbols) compared to SVF-coated meshes (in red symbols) for every animals (**a**). Illustration of the intensity of the vascular flow mapped using the doppler system, given as example for the rat 1 with the corresponding macroscopic observation of the two groups. The dash lines delineate the area of the abdominal wall covered with the mesh (**b**). Illustration of H&E staining revealing vascularization of fibrin-coated mesh (**c**, day 21, the black arrows point the vessels and the star denotes the mesh cross section), along with the quantification of vessel cross sections (**d,** $ denotes significance between control group day 10 versus day 21). Illustration of 3D reconstruction of perfused vascular branches using high-resolution microCT of SVF-coated mesh (**e**) with the resulting ratio volume vessel to volume tissue expressed in percentage for three animals of each group per time point, calculated form the middle of the explanted graft (1 cm^2^, **f**). The mean values for both group at both time point is shown with the bars with a grid pattern, with $ denoting significance between SVF and fibrin-coated mesh at only day 21

The degree of vascularization of the meshes was assessed through LDI, histology, and high-resolution tomography (Fig. 5). Doppler analysis conducted at day 21 showed that there was a significant difference in terms of degree of vascularization amongst the six rats, independently on the nature of the implanted group. Nevertheless, no significant difference was noted for the SVF-coated meshes compared to the control meshes (Fig. 5a, b). H&E histological staining revealed as well that SVF-coated and fibrin-coated PP meshes were prone to high vascularization. No statistical significance was noted for the number of vessel cross sections quantified for the fibrin versus SVF-coated group, at day 10 and 21, which corroborated LDI quantification (Fig. 5c, d). Nonetheless, the increase of the vessel density over time was significant for the fibrin group (Fig. 5d). The quantification of the vessel volume/tissue volume revealed a decrease between day 10 and day 21 only for the SVF-coated group, with difference becoming significant compared with the fibrin group at day 21 (Fig. 5e, f).

**Fig. 6:**
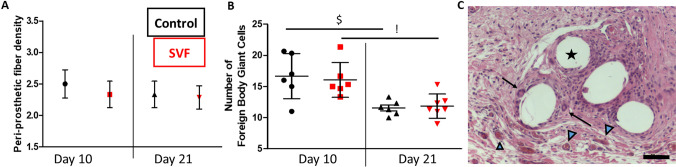
Quality of the mesh integration. Appreciation of the fiber density of the peri-prosthetic neoformed tissue (**a**, 1-very loose; 2-loose; 3-dense; 4-very dense, no statistic difference was observed between the groups). Intensity of the immune response was analyzed by counting the foreign-body giant cells (no statistical difference was noted between control and SVF-coated group, but only between day 10 and day 21 for control- ($) and SVF-coated group (!), **b**). H&E staining of SVF-coated group day 21 (scale bar represents 50 µm, black star denotes the mesh cross section, black arrows point the foreign-body giant cells and blue triangles show the vessels, **c**)

The histology corroborated the macroscopic observations summarized Table 1, and the H&E staining showed that the peri-prosthetic tissue, in terms of fiber density did not change significantly between the groups (Fig. 6a). As expected, a chronological attenuation of the intensity of the immune response reported by the foreign-body giant cell quantification was observed for both control- and SVF-coated mesh, from day 10 to day 21 (Fig. 6b). Vascularization and presence of FBGC close to the surface of the mesh is illustrated in Fig. 6c.

**Fig. 7:**
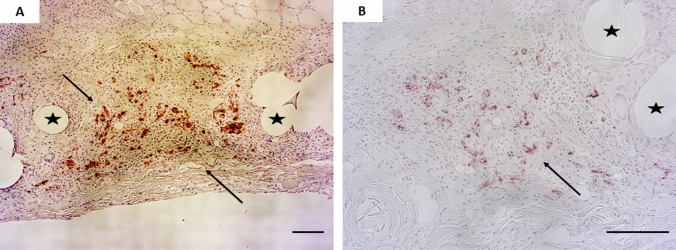
Human SVF cells were detected 10 days post-surgery at the implantation site. Positive anti-human mitochondrial immunostaining was detected by the brown color (denoted with the black arrows) between the filaments of the mesh (denoted with the black stars). Scale bars represent 100 µm

In our study, anti-human mitochondrial immunostaining revealed that SVF cells were still present in the hernia defect 10-days post-surgery (detection in three out of seven rats, Fig. 7). No positive staining was detected for the animals euthanized after 21 days, meaning that, either the human SVF cells died from day 10 to day 21, or that they migrated out of the hernia defects.

## Discussion

Recent advances in tissue engineering highlight the advantages to combine cells with the implanted materials to accelerate tissue repair [2]. This is particularly relevant in abdominal wall hernia repair where preclinical studies tend to demonstrate that coating meshes with cells brings advantage in terms of tissue integration and mitigates immune response against the foreign materials [4–6, 8, 16]. Preclinical investigations showed as well that coating meshes with cells prevented adhesion formation [17, 18]. More recently, clinical case report confirmed that (1) coating a prosthesis with patient’s cells improved fascial healing, and that (2) this is a feasible strategy for challenging elective procedures, such as high-grade incisional hernia, wound infection, or surgical acts on obese patients [19]. Cell seeding could also be envisioned for the preparation of “cellular 3D mesh-like scaffolds” to repair and “fill” large defects not only in classical hernia repair, but also for reinforcement of the pelvic floor after abdominoperineal resection for instance.

In such combination, the selection of appropriate cell source is a key parameter for a successful tissue reconstruction, and due to the invasiveness to isolate bone marrow (BM)-MSCs, other options must be investigated. Adipose tissue, considered a biological waste product, can be easily obtained from the patients and contains numerous cells, other than adipocytes, with great interest in regenerative medicine [15, 20]. To overcome the regulatory bottleneck of enzymatically-processed biological products, we have developed a protocol based on mechanical disruption of the fat which does not require the utilization of any enzymes or chemical compounds (Fig. 2). Previous experiments from our institute have already shown that SVF cells isolated from adipose tissue using “gentle procedure” preserved a high degree of viability and can easily proliferate once embedded in a fibrin clot [11]. In another study, grafts of chitosan with SVF cells have been tested to repair a full-thickness defect created on the wall of the colon. Compared to cell-free control graft, the presence of SVF permitted to decrease the intensity of the fibrotic reaction and allowed for the regeneration of muscular fibers [21]. Nevertheless, to the best of our knowledge, no translation toward the field of hernia has been undertaken. For this reason, we proposed here to use a fibrin gel with SVF still embedded in their natural ECM niche as biological coating for PP mesh. Other vehicles for cells have been reported for mesh coatings, with good outcomes using for instance platelet-rich plasma (PRP) [19]. Nevertheless, as PRP is naturally enriched with numerous growth factors (PDGF, VEGF, TGF, FGF, etc.), it would have been difficult in our study to decipher the biological role of SVF in a coating based on the combination PRP–SVF. This motivates the usage of fibrin, which is well known in the field of wound healing and sealant for hernia meshes [22–24].

This fibrin-loaded SVF microfragment was shown to be a suitable coating for hernia meshes. This animal model was selected from Majumder et al., who demonstrated that MSC-coated meshes induced optimal tissue integration when placed as onlay versus intraperitoneal underlay [25].

We presented in this study a new methodology to assess and quantify the degree of vascularity of the graft in 3D, using tomography on perfused explants. The presence of contrast agent (i.e. sulfate barium) followed by the incubation of the grafts in Lugol solution allowed to give a clear and strong contrast between the vessels, the peri-prosthetic tissue and the mesh. Such methodology is relatively simple, inexpensive, and can be used for the 3D appreciation and quantification of implants’ vascularization, even outside the research field of abdominal wall hernia repair. Vascularization is a result of the short-term inflammatory reaction (first few weeks) and impacts on the long-term tissue remodeling and integration of the mesh (following few months up to years) [3]. The level of angiogenesis in the early healing phase correlates with the degree of inflammatory reaction [26]. After the initial growth of blood vessels activated by proangiogenic factors released by the inflammatory cells, tissue healing is chronologically characterized by a proliferative and a remodeling phase. Those phases are associated with a decrease of the level of vascularization of the wound, until returning to normal level. Preclinical study using rats with onlay model presented by Wolf et al., demonstrated that post-surgical vascular response peaked between 7 and 35 days, which reaction was attenuated using an ECM-based coating [27]. In our study, the presence of SVF cells slightly, but significantly, attenuated the growth of vessels from day 10 to day 21 compared to control group (Fig. 5d, f). This can potentially be a sign of an accelerated maturation of the capillary network, but longer time points are needed to assess if this decreased level of vascularization correlates as well with a more robust vascular integration of the prostheses.

Preservation of transplanted cells at the site of implantation is one of the greatest and redundant challenges to have coating with a sustained biological activity. Lin et al*.,* detected human SVF aggregates following subcutaneous implantation in nude mice after up to 3 months [28]. In our study, anti-human mitochondrial immunostaining revealed that SVF cells were still present in the hernia defects 10-days post-surgery (detection in three out of seven rats, Fig. 7). No positive staining was detected for the animals euthanized after 21 days, meaning that, either the human SVF cells died from day 10 to day 21 in the animals, or that they migrated out of the hernia defects. Due to the great capability of SVF cells to release paracrine factors which can stimulate cells recruitment and tissue repair away from implantation site [29], the absence of the transplanted SVF cells from the wound bed can still be responsible for a biological activity. For instance, SVF cells have been shown to contain anti-inflammatory cues, suppressing cytokine secretion and activating immune cells following transplantation [13]. As a consequence, conducting preclinical tests using a larger and immunocompetent animal model, on which autologous SVF transplantation can be undertaken would be of interest to assess specifically the immunological response and degree of foreign-body reaction.

## Conclusion

The utilization of minimally manipulated cells originating from autologous biological tissues has raised tremendously in the field of tissue engineering and regenerative medicine. Amongst the emerging trends in stem cell therapies, SVF cells are assessed in numerous preclinical and clinical trials. In this study, we developed a workflow allowing to process liposuction products to obtain SVF cells without the need of any enzyme-based protocols. The SVF can be integrated in a temporary carrier such as fibrin gel, and be used as biological mesh coating ready to be transplanted, without the need for further cell processing. The first attempt to repair hernia defects using SVF-coated meshes presented in this publication revealed that the cellular grafts were well tolerated by the animals and decreased the short-term vascular growth. Further investigations are required to validate that SVF cells help in the establishment of an appropriate vascularization and integration of the polypropylene graft in a long-term experiment.
